# Supplementary material for: Are carbon emissions trading and green financial instruments synergistic? -Comprehensive quantitative research based on content analysis
Source: PLoS One. 2024 Mar 7;19(3):e0298601. doi: 10.1371/journal.pone.0298601 (PMC10919601; doi:10.1371/journal.pone.0298601)
Supplement: S1 Data — (ZIP) [file pone.0298601.s001.zip › Data/地级市Z_F工作报告.zip!/╦─┤¿/╣π╘¬╩╨/└┤╫╘╣1⁄2╓┌║┼í╛┬φ┐╦╩2╛▌═°í┐_2015.docx]

各位代表：

现在，我代表市人民政府向大会报告工作，请予审查，并请市政协各位委员提出意见。

2014年工作回顾

过去一年，面对复杂严峻的宏观经济形势，在市委的坚强领导下，在市人大、市政协的监督支持下，我们始终保持专注发展定力，迎难而上，克难攻坚，完成了全年经济社会发展主要目标任务，全面建成小康社会打下坚实基础，全面深化改革实现稳步开局，全面推进依法治市开启新征程，全面落实从严治党取得新进展。全市实现地区生产总值566.19亿元，增长9.2%，增速居全省第8位，比上年提升3位;地方一般公共预算收入34.78亿元，增长14.2%；城镇居民人均可支配收入20547元，增长9.8%；农民人均纯收入7202元，增长11.8%；居民消费价格涨幅1.9%。

一、转型升级步伐加快，经济保持平稳增长

综合施策稳增长。在认真贯彻落实国家、省稳增长系列政策措施同时，及时推出稳增长“二十四条”，出台了系列支持实体经济发展的政策措施，从多方面稳定经济增势。深入开展“项目攻坚年”活动，加快实施夯基础、补短板、增后劲、惠民生的重大项目88个，完成投资224.63亿元，万龙路等8个重大项目竣工，上石盘电航综合枢纽等39个重大项目加快推进，广元天立国际学校等16个重大项目开工建设，大唐广元新建电厂等25个重大项目前期工作进展顺利。完成全社会固定资产投资561.74亿元,增长3.8%。产业投资持续增加，完成工业投资190.1亿元，增长6%；完成第三产业投资332.77亿元，增长3.1%，其中房地产投资83.32亿元，增长42%。实现民间投资283.43亿元，对投资增长贡献率41.5%。消费拉动明显增强，组织3000多家企业参加市场拓展“三大活动”，成功举办女儿节、秦巴山区年货节等地方性节会，积极培育新的消费热点，实现社会消费品零售总额264.23亿元，增长12.6%。

着力调整产业结构。坚持在扩增量中调结构，三次产业结构调整为17.4:47.7:34.9。工业加速转型升级，实现全部工业增加值231.03亿元，增长9.1%，其中规模以上工业增加值增长9.8%。继续推进煤炭企业兼并重组，关停小煤矿7处、非煤矿山77处，淘汰落后和化解过剩产能企业51户。新培育亿元以上企业29户、规模以上企业50户，中石化元坝气田采输和净化厂、兴能新材料等一批重大工业项目竣工投产。推动广元国家级经济技术开发区加快发展，苍溪县工业集中发展区创建为省级经济开发区，剑阁经济开发区创建为全省第三批小企业创业示范基地、省级高新技术特色产业化基地。新建标准化厂房9.8万平方米，新增工业园区9.2平方公里。农业稳步发展，实现农业增加值98.57亿元，增长4.2%。粮油生产实现“八连增”。新建优势特色农业产业基地30.5万亩、现代农业园区10个。服务业加快发展，实现增加值197.68亿元，增长11.5%。新增限额以上批零住餐单位57个，万达广场、川北国际商贸物流港等项目加快推进。新增国家4A级旅游景区2个、省级旅游度假区2个、省级生态旅游示范区2个，剑门关-翠云廊创建国家5A级旅游景区通过国家专家组暗访，成功创建唐家河国家级生态旅游示范区，成功举办2014年中国·四川国际文化旅游节。全年接待游客2769.7万人次，实现旅游总收入158.71亿元，增长41%。金融机构各项存款余额1006.64亿元，增长7.6%，各项贷款余额486.33亿元，增长17%；保费收入增长21.5%；四板挂牌展示企业总数达17户。

大力实施“创新驱动”战略。深度推进“厅市会商”“市校合作”和“校企合作”，签订科技合作协议73项，新建省、市级企业工程技术研究中心（实验室）12个；新建院士（专家）工作站6个，培育孵化单位16个。申请专利939件，增长30%。取得重大科技成果39项，获得全省科技进步奖7项，元泰达超大规格闭孔泡沫铝全自动生产技术获全省科技进步一等奖。实施科技成果转化项目153个，实现产值165亿元，增长10%。开发市级重点新产品30个，飞亚新材料聚芳醚醚腈和零八一大尺寸非晶态软磁合金磁芯获得国家重点新产品立项，新增四川名牌产品27个，新增国家级高新技术企业9户、省级创新型企业4户，实现高新技术产业总产值123亿元，增长9.8%。

二、统筹发展扎实推进，城乡面貌不断改善

以“三园一区”为载体的贫困山区统筹城乡发展模式得到省统筹城乡试点成果评估组的充分肯定。城乡规划体系进一步完善，中心城区控详规实现全覆盖，城镇控详规覆盖率60%。启动中心城市总体规划中期评估，完成基本生态控制线等专项规划，全面完成县区总体规划修编，完成221个乡镇、879个新村规划编制。城乡建设提质加速，完成建设总投资164亿元，增长10%，实现建筑业增加值38.91亿元，增长12.1%；质量安全监管更加有力，澳援大桥获得省工程质量最高奖“天府杯”金奖。建成水柜路、瓷莲路等城市交通干道，中心城区“三横九纵”道路交通体系初步形成，打造了廊桥步行街、滨江步行道等一批城市休闲配套设施，老城人防、市城区部分街道雨污分流等工程建成使用，完成利州东路路灯改造工程，城市品位显著提升。棚户区、旧城区改造深度推进，三江新区、万源新区、零八一产业新城、大荣新区等城市新区加快建设，全市新增建成区8平方公里。全市城镇化率39.33%，比上年提高1.53个百分点。“百镇建设行动”扎实推进，城镇功能日趋完善，吸纳农业转移人口7890人，旺苍三江镇等5个镇入选第三批试点镇。城乡环境综合治理和爱国卫生运动深入推进，城市管理水平进一步提高，成功创建国家卫生城市。新建幸福美丽新村200个、新型农村社区7个、农村聚居点110个，改造和新建民居3.34万户。城乡交通发展提速，国省干线联网畅通，农村公路改善等“八大交通专项工程”有序推进，新建农村公路1711公里、路侧护栏1570公里，改造提升汽车客运站10个，建成渡改人行桥14座。新建农田水利渠系2083公里，苍溪乐园、青川曲河、朝天双峡湖3座中型水库加快建设，亭子口灌区一期、武引灌区二期等重点灌区工程建设有序推进。大力实施土地整理项目，建成高标准基本农田33.95万亩。解决了11.2万农村人口和农村学校4万师生饮水安全问题。

生态文明建设加快推进。国家低碳城市试点工作纵深推进，成功申报创建国家新能源示范城市，成功创建省级环保模范城市。森林覆盖率54.6%。创建省级生态乡镇33个、市级生态村65个，新建农村户用沼气1.6万口，新建绿道和生态廊道60公里。“气化广元”持续加快，城镇气化率78.5%。全市单位生产总值和单位工业增加值能耗分别下降6.69%、11.92%，完成总量减排省定目标。狠抓重点流域水环境综合整治和饮用水源保护，取缔白龙湖网箱养殖工作有力推进，白龙湖纳入国家良好湖泊保护和省重点小流域污染防治规划；县级以上城镇集中式饮用水源地水质全部达标。市城区环境空气质量优良天数占比99.7%，环境空气质量持续居全省前列。

三、改革开放全面推进，发展活力持续增强

以放活土地经营权为重点，加快农村改革步伐，以“七权同确”为主要内容的农村产权制度改革经验在全省交流，“1+3”广元特色新型农业经营体系初步形成。积极推动投融资体制改革，落实省政府投资项目核准目录，鼓励社会资本投资重大项目。一元化户籍制度改革取得实质性突破。“营改增”试点不断推进，税收分享体制改革基本完成，财政绩效预算管理改革取得新进展。启动了公车改革。农村金融创新步伐不断加快，苍溪县农村信用社改制为农商银行。完善推动民营经济发展的体制机制，实现民营经济增加值312.1亿元，增长10.7%，对GDP增长贡献率达62.5%。稳步推进价格改革，完成市城区供水阶梯水价和农村客运票价改革。企业注册资本实缴改认缴、年检改年报、信用信息公示等商事制度改革全面实施。就业和社会保障、人事人才、教育、科技、文化、医疗卫生等领域改革有力推进。

全市实施招商引资项目687个，引进到位市外资金370亿元，其中到位省外资金193亿元。大力推进区域合作，与乌鲁木齐市签订友好城市战略合作协议。成功举办“九广合作”活动，签订帮扶协议13个、投资项目协议6个。深度推进与中石化战略合作，实现广元天然气净化有限公司在广元注册。成功举办广元-马来西亚投资贸易座谈会，达成8项意向合作协议，实现海外招商零突破。

四、民生持续有效改善，发展成果惠民共享

全面完成“十项民生工程”和32件民生实事。全年民生支出131亿元，占地方一般公共预算支出比重68%。积极创建省级创业型城市，扶持创业1.36万人，城镇新增就业3.85万人，城镇登记失业率3.9%。转移输出农村劳动力97.8万人，实现劳务总收入139.95亿元。启动了“全民参保登记计划”试点，建立了城乡居民养老保险丧葬补助制度、大病医疗保险制度，社会保险经办管理实现“一卡通”，医疗保险实现全省范围内异地及时结算。各项社会保险累计参保272.44万人次。城乡低保对象实现应保尽保。为2.44万名重度残疾人发放护理补贴1570万元。开工建设各类保障性住房1.04万套，向农民工提供公共租赁住房787套，改造农村危旧房1.35万户。四个“三位一体”精准扶贫经验在全省推广，全年减少农村贫困人口5.67万人,实现易地扶贫搬迁1010户3750人。10万新老库区移民安置和后扶工作有序推进。实施地质灾害治理工程80处，实现地质灾害防治隐患点零伤亡。完成789个自然村地面数字电视建设，本地电视节目覆盖率提高10%。全面实施“十大救助制度”，建成全国综合减灾示范社区5个，新建城乡日间照料中心111个、城乡居民养老服务点227个、农村幸福院116个。

统筹发展社会事业。学前教育三年毛入园率达82%，残疾少年儿童入学率达80%；推进义务教育均衡发展，学生营养改善计划惠及18.6万农村学生；大力推进高中教育优质特色发展，高考本科上线稳定在万人以上；现代职业教育加快推进，中职学生高职对口单招和高考录取人数稳步增长，技能比赛成绩居全省前列；川北幼专和四川信息职业技术学院办学影响力不断提升，四川健康职业技术学院创建纳入省高校设置规划。县级公立医院综合改革稳步推进，14家县级公立医院全面取消药品加成；城乡居民健康档案规范化电子建档率99%，省级标准化乡镇卫生院达标率100%、村卫生室达标率99.44%；创建全国农村中医药工作先进市取得新进展；计划生育“两奖两扶”政策全面落实，启动实施“单独两孩”政策，人口自然增长率3.26‰。苍溪、旺苍、剑阁通过全国文化先进县复查，13个乡镇被评为四川省民间文化艺术之乡；5件作品获全省“五个一工程”奖；成功创建中国楹联文化城市；设立并评选首届广元文学奖；万安博物馆实现对外开放；考古调查取得重大发现，初步完成蜀道申遗文化遗产预备清单编制；全民健身运动蓬勃发展，省第十三届运动会、第九届残运会、第四届特奥会筹备工作全面启动；顺利通过国家二类城市语言文字工作评估达标验收；市档案馆创建成国家一级综合档案馆。

审计、统计、防震减灾、测绘、气象、水文、科普、工会、妇女儿童、青少年、老龄、地方志、新闻出版、红十字、慈善、外事侨务、港澳台、民族宗教等工作都取得了新成绩。

五、社会治理全面加强，社会保持和谐稳定

不断加强和创新社会治理。四级网格化社会管理服务体系不断完善，社区管理服务能力全面提升，建成新型农村社区363个。“法律七进”活动扎实开展，“六五”普法深入推进，创建省级“法治城市”工作全面开展。“五建五强”群众工作法全面推广，服务群众平台体系进一步健全。深入开展“信访工作走基层行动”，大批积案难案得到妥善化解，全市信访总量持续下降，信访形势明显好转。社会稳定风险评估工作有力推进，条块结合、纵横联动、覆盖全域的评估体系基本形成。

深化“平安广元”建设。严厉打击各类违法犯罪活动，未发生有重大影响的恶性刑事案件和治安案件。在全省公安机关政风行风第三方测评中，我市公众安全感和满意度名列全省第一。全面推进安全生产监管体制改革，扎实开展“道路交通安全综合整治攻坚年”和“企业安全生产主体责任落实年”活动，深入推进安全社区建设，安全生产基层基础进一步夯实；狠抓各行业安全生产，未发生重特大安全生产事故，安全事故控制指标考核居全省第2位。加强食品药品、农产品安全监管，未发生大的食品药品安全事故，获评省级农产品产地无公害化市，成为全省首个农产品质量安全监管示范市。加强工商、质量和金融监管，市场秩序进一步规范。应急管理、国家安全、保密、消防、人防、国防动员、民兵预备役、双拥等工作全面加强。

六、政府自身建设全面加强，服务能力不断提升

过去一年，我们始终坚持重大行政决策法定程序，坚持依法科学民主决策，加快法治政府建设。深入推进行政权力依法规范公开运行，加强行政权力监督，规范行政执法行为。巩固完善政务服务体系，优化办事流程，政府服务效能进一步提升。深化行政审批制度改革，建立行政职权目录清单，清理出市本级行政权力6325项、行政许可227项、行政许可前置条件425项，清理出行政事业性收费95项、经营服务性收费87项；完成涉及审批服务的中介机构信息采集、行业协会学会摸底和脱钩方案制定。深入推进市县区政府机构改革，整合卫生计生、农业畜牧水产、食品药品等机构职能，建立形成了大卫生、大农业、大旅游、大文化管理机制和市场监管体系，事业单位分类改革稳步推进。认真开展政府投资项目招投标秩序专项整治、重点工程施工环境专项治理，经济发展软环境进一步优化。深化政府信息公开，开通广元政务微信、微博公众平台，推进了决策、执行、管理、服务和结果公开。办理人大代表建议140件、政协委员提案264件，办结率、满意率均达到100%。严格执行中央“八项规定”、国务院“约法三章”、省委省政府“十项规定”和市委“六个带头”，集中开展了“作风效能八大整建行动”、正风肃纪“9+3+X”突出问题和“庸懒散浮拖”问题专项整治，持之以恒纠治“四风”，严厉惩治腐败行为，政府系统作风建设和廉政建设进一步加强。

各位代表，过去一年，是近年来我市发展面临困难最多、压力最大的一年，经济社会发展能取得这样的成绩，是市委科学决策、坚强领导的结果，是全市人民齐心协力、不懈奋斗的结果。在此，我代表市人民政府，向全市人民，向人大代表、政协委员，向各民主党派、工商联、无党派人士和人民团体，向驻广部队、武警官兵、政法干警和民兵预备役人员，向所有关心支持广元发展的同志们、朋友们，致以崇高的敬意和衷心的感谢！

各位代表，总结去年工作，我们也清醒地看到还存在一些困难和问题：一是部分经济指标增速回落幅度较大，投资增长乏力，新的消费热点不多，出口困难加剧，稳增长难度加大。二是产业结构不优，一些传统产业持续萎缩，新落地的重大新兴产业项目不多。三是生产要素成本上升，中小企业融资难、融资贵问题突出，生产经营困难增多。四是一些改革举措尚未完全落地，妨碍发展的体制机制障碍亟待破除。五是对照全面实现小康的目标,基础设施、公共服务、改善民生、扶贫攻坚等任务还十分艰巨，群众对医疗、养老、教育、食品安全等还有不少不满意的地方。六是在政府工作和自身建设方面，有些决策部署和工作措施还没有落到实处，少数政府机关工作人员仍然存在不作为、乱作为和“庸懒散浮拖”等不良现象，一些领域腐败问题仍然存在，依法行政还需加强。对此，我们将以对党和人民高度负责的态度，直面问题，勇于担当，认真研究解决。

2015年主要目标任务和重点工作

今年政府工作的总体要求是：认真贯彻落实党的十八届三中、四中全会和省委十届四次、五次全会精神，全面落实市委六届七次、八次、九次全会部署，按照“四个全面”战略布局，主动适应经济发展新常态，坚持总体发展思路不动摇，坚持“稳中求进、好中求快”工作总基调，以提升经济发展质量和效益为中心，深入实施“五大发展战略”，加快转型升级，全面深化改革，强化开放合作，加强民生保障，深化依法治市，推动经济持续健康发展、社会和谐稳定。

主要预期目标建议：地区生产总值增长8.5%；规模以上工业增加值增长9.5%；全社会固定资产投资增长3.3%；社会消费品零售总额增长12%；地方一般公共预算收入增长8.5%；城镇居民人均可支配收入增长11%，农民人均纯收入增长12%；居民消费价格涨幅控制在3%左右；城镇登记失业率控制在4%以内；人口自然增长率控制在4.5‰以内；城镇化率提高1.5个百分点；森林覆盖率达55.25%；全面完成节能减排目标任务。

今年经济增速预期目标高于全国1.5个百分点、全省1个百分点，但低于去年全市的增速。作出这样的安排，主要基于三个方面的考虑：第一，主动适应经济发展新常态的重要要求。经济新常态是中央对我国经济发展阶段新变化作出的重大战略判断。广元和全国全省基本一致，总体上也已进入速度换挡期，必须主动适应新常态，向改革要动力、要活力，坚持转方式、调结构，促进提质增效、可持续发展。第二，推进同步建成全面小康的迫切需要。到2020年要与全国全省同步建成全面小康，这是全市人民的共同愿望，也是我们为之奋斗的目标。但目前我市人均GDP、城乡居民收入都低于全国全省平均水平，有30多万人还没有脱贫，这就要求我们务必坚持比全国全省快一些的发展速度。从多方面看，只要我们充分挖掘发展的空间和潜力，用好国家推动“一带一路”和长江经济带发展战略、实施秦巴山片区区域发展与扶贫攻坚规划等多重发展机遇，我们完全有条件、有信心保持高于全国全省的平均增速。第三，正视困难风险的清醒估价。当前，国际国内经济形势、宏观政策、市场需求、发展动力、资源环境等方面已发生很大变化，无法继续支撑过去那样的高增长，短期波动性会增强，经济、社会风险因素叠加显现，这就要求我们既要抓经济发展新动力的形成，又要着力抓综合风险的防控，坚持在“量力而行、尽力而为”的原则下把握好发展的预期和节奏，合理确定预期目标。

新的一年，我们将充分调动一切积极因素，始终保持专注发展定力，在“抓紧转”的同时，更要“加快赶”，为利长远而稳当前，奋力完成今年发展目标和“十二五”规划确定的各项目标任务，同时编制好“十三五”规划，谋划好未来五年的发展蓝图。重点抓好以下工作：

一、加快工业转型升级，努力实现提质增效

以提高质量和效益为中心，坚持在盘活存量中转方式，在扩大增量中调结构，走内涵式发展路子。启动实施“工业升级三年行动计划”。把传统产业改造提升摆在突出位置，实施投资过亿元技改项目60个，完成技改投资120亿元。积极淘汰落后和化解过剩产能，继续推进煤矿兼并重组和小煤矿关停。突破性发展战略性新兴产业,以五大高端成长型产业和28个重大工业项目为重点，扶持一批拥有自主知识产权、具有较强市场竞争力的战略性新兴产品，推动新兴产品规模化、产业化，力争培育战略性新兴产品20个以上。支持中小微企业加快发展，培育新进规模以上企业45户。以新增产值千万元以上增长点企业为重点，实施领导联系、问题台账和销号管理制度，积极争取直购电、丰水期富余电量消纳等用电优惠政策，加大对用气大户直供的协调力度，加强交通运输协调，降低企业综合物流成本，确保工业经济稳定增长。

创新园区建设投融资模式，鼓励和引导民间资本参与园区建设，加强园区基础设施建设，强化集约节约用地，力争园区用地产出率增幅提高3个百分点。加大园区项目引进和建设力度，促进更多项目落地和竣工投产。加快国家级、省级经济开发区发展，积极推进朝天区创建省级经济开发区、利州区创建省级高新技术产业园区，大力推进零八一机电电器产业园、剑阁兴能新材料产业园、朝天七盘关国际石材城、旺苍黄洋建材产业园等特色园区建设，不断做大园区规模，促进产业集中、集聚、集约发展，力争工业集中度达到72%。

创新人才引进、培育、使用政策机制，加快产学研用协同创新发展，培育市级以上企业工程技术研究中心（实验室）10个，建立院士（专家）工作站5个，培育孵化单位8个。对接产业链谋划创新链、人才链、资金链，突出关键技术攻关、科技成果转化、产品创新与高新技术企业培育，实施科技成果转化项目140个，培育申报国家高新技术企业10户、省级创新型企业5户，力争高新技术产业总产值达130亿元以上。大力推进工业品牌战略和企业标准化建设，支持企业创建国家级、省级品牌。探索开展智能制造试点示范，推动工业化和信息化深度融合，促进结构优化升级。进一步激励科技人才、大学生创新创业，形成尊重知识、尊重人才的浓厚氛围。

二、强化项目投资拉动，保持投资稳步增长

按照“四个一批”要求，狠抓107个重大项目，确保完成投资220亿元以上。力争国道108线下寺至普安段改线、智能手机平板电脑及其零组件生产等一批重大项目竣工投产；加快推进兰渝铁路、西成客专等一批在建重大项目，完成投资153亿元以上；新开工广元至平武高速公路、国道108线鸭浮岩至纺织大道等一批重大项目，完成投资55亿元以上；积极做好广巴铁路扩能改造、广元国际生态休闲养老产业园等一批重大项目前期工作。主动对接国家、省规划和政策投向，努力谋划和储备一批对长远发展具有战略意义的重大项目，确保项目滚动储备1万亿以上。

稳定和扩大政府公共投资规模，积极对接国家、省重大工程包，加强交通、水利、生态环保、能源等领域的项目建设，增加公共产品有效供给。加快推进政府融资平台转型发展。严格落实“五个一律”要求，切实发挥财政资金杠杆作用，撬动社会资本参与市政设施、保障性安居工程、文化、教育、卫生等重点领域建设，充分激发民间投资活力，实现民间投资300亿元以上。

继续实行重大项目“四个一”推进机制，完善项目工作目标考核机制。切实抓好征地拆迁，保障项目建设用地。创新拓展融资渠道，积极搭建银政、银企合作平台，有效破解企业生产、项目建设融资难题。进一步优化发展软环境，为项目实施创造良好条件。

三、转变农业发展方式，促进农民增收农业增效农村发展

在巩固提升粮油、生猪、劳务“三大传统百亿产业”的基础上，以市场为导向，强化政府引导和支持，加快发展优势特色产业，突破发展木本油料、红心猕猴桃、茶叶烟叶、蔬菜、土鸡“五大特色百亿产业”，发展核心基地面积15万亩。完善特色产品标准体系，持续推进绿色农业发展，提升“广元七绝”、 剑门关土鸡、广元纯黄茶、广元高山露地蔬菜品牌效应。着力构建以家庭农（林）场为主体，以“农民专业合作社+产业发展领军人+社会化服务超市”为依托的“1+3”广元特色新型农业经营体系，培育家庭农（林）场500个，发展农民专业合作社150个，建立社会化服务超市166个，培育销售收入过亿元龙头企业7家。

统筹推进“三大建设”。按照“物的新农村”和“人的新农村”建设齐头并进的要求，启动建设200个幸福美丽新村、300个新村聚居点、1000户农村廉租房和7个新农村综合体（新型农村社区），改造农村危旧房1.4万户。加快“1+6”村级公共服务体系建设。切实加强传统文化村落、古民居、乡土特色建筑保护，抓好国家级和省级生态文明乡村创建。持续抓好现代农业园区综合建设，新建7个、提升7个。加强农田水利基本建设，切实做好永久基本农田划定工作，建设高标准基本农田34.62万亩，新建和整治渠系400公里；持续推进城乡交通一体化建设，大力实施农村公路“三年攻坚行动”，新建农村公路800公里、路侧护栏1490公里，完成渡改桥9座、溜索改桥10座，新建汽车客运站10个。解决11万农村人口和农村学校师生饮水安全问题。新建农村户用沼气1.23万口。

充分挖掘特色种养业助农增收潜力，加大农产品产地初加工和深加工支持力度，积极发展休闲农业、创意农业和乡村旅游业，持续增加农民家庭经营性收入。认真落实好各项强农惠农政策，增加农民转移性收入。加强劳务品牌建设，强化技能培训，全年转移输出劳动力97万人，实现劳务收入140亿元以上，稳定增加农民工资性收入。积极探索农村产权抵押、担保和转让改革试点，增加农民财产性收入。深化提升四个“三位一体”精准扶贫机制，在北部山区、移民安置区、边远山区持续实施各类扶贫开发、移民安置和后扶项目，推进152个贫困村脱贫解困，全年减少农村贫困人口6万人以上，实现易地扶贫搬迁2362户0.91万人。

四、加快发展现代服务业，努力扩大消费需求

立足资源、生态、区位、基础设施等比较优势，重点扩大旅游休闲、绿色生态、健康养生、家政、信息、住房、文化体育、教育卫生等领域消费。组织企业参加市场拓展“三大活动”，举办好女儿节、秦巴山区年货节等地方性节会。优化城市商业中心业态结构，提升商品消费服务水平。支持农产品批发市场实施信息化提升工程，加快“七绝商城”等农产品电子商务平台建设。支持城乡消费设施建设，推进电子商务进农村综合示范，构筑城乡现代流通体系。

突破性发展旅游产业。统筹推进剑门蜀道、唐家河、米仓山、嘉陵江“四大精品旅游区”和曾家山中国西部养生基地、乡村精品旅游区建设，剑门关-翠云廊创建成国家5A级旅游景区，加快推进蜀道申报世界自然与文化遗产工作。加强旅游与文化、体育、商贸、康养、新型城镇化、新农村建设的深度融合。积极推进文化旅游产品创新，打造游客参与体验项目。大力发展乡村旅游，打造一批各具特色的旅游小镇和度假村落。积极推动旅游管理体制改革创新，探索推进旅游企业资本重组，推进有条件的旅游企业上市。开展“旅游服务质量提升年”活动，推进全国旅游标准化示范市创建工作，全面提升旅游服务质量。大力实施旅游整合营销，办好剑门蜀道三国文化旅游节。力争实现接待游客3000万人次、旅游总收入195亿元、门票收入2亿元。

抓好川北国际商贸物流港等重点项目建设，打造现代物流集聚区。加强金融生态环境建设，推动银政企深度合作，提升金融服务水平,新增贷款70亿元以上。加快建设广元电子商务物流产业园区。大力推进“智慧广元”和“宽带乡村”建设。积极发展健康养生、康复医疗、健身休闲、养老服务等业态和经营模式，大力发展居家养老和社区养老服务。深入实施服务业“双百工程”，力争新进限额以上商贸流通企业30户。扎实抓好市城区“四大商圈”和特色商业街区建设，加快实施万达广场、图腾·海博春天等一批在建重点商贸项目。加快建材、家居、汽车、五金机电等专业市场建设。实施好汽车客运站改造提升工程，加快雪峰、昭化货运枢纽站建设。积极把广元港推向市场，尽快形成水上运力。认真贯彻落实促进房地产发展政策措施，促进房地产市场健康发展。

五、深入推进新型城镇化，促进城乡一体化发展

加快城市基础设施建设，完善“三横九纵”道路交通体系，强力推进北二环、滨河南路万源至龙洞扁段和滨河北路红星公园至荣山段等重点项目建设，新开工西二环、下西大道等交通主干道路网项目；强力推进城中村、背街小巷、老旧院落、商业中心改造提升工程。加大棚户区和危旧房改造力度，开工建设保障性住房4537套，改造城镇危旧房棚户区4761户，切实抓好棚户区还建房建设。加快大荣新区、三江新区、苍溪江南新区、旺苍大中坝新区、剑阁剑门新区、青川竹园新区、朝天草房沟新区等城市新区建设，力争全市新增建成区5平方公里。加强城市供水、供气、供电、地下管网、防洪防涝设施等建设。继续实施“百镇建设行动”，加大小城镇建设力度，突出抓好14个国家重点镇、15个省级试点镇建设，力争完成村镇建设总投资20亿元。着力打造一批特色鲜明、优势明显的工业强镇、旅游名镇、特色农业镇、商贸服务镇。规划建设一批基础设施和公共服务设施健全、产业相对聚集的新型城乡社区，力争城镇社区服务设施综合覆盖率达94%。大力支持建筑业转型升级、加快发展，力争增加值增长11%。

坚持以“三园一区”为载体，继续推进城乡规划、基础设施建设、产业发展、公共服务和社会治理“五个统筹”，加快城乡统筹发展。加快城镇基础设施向农村延伸，推进城乡基本公共服务均等化，促进城乡要素平等交换和公共资源均衡配置。开展市县“多规合一”规划编制，做好中心城市、县城控详规维护，加快乡镇总规及建制镇控详规编制。注重文化传承、生态保护和功能优化，合理确定城镇终极规模，促进城镇集约、智能、绿色、低碳发展。切实提升县城和中心镇产业、人口承载能力，方便农民就近就地城镇化。落实好城市“四定”网格化管理制度，不断提高城市管理的法制化、规范化、精细化和信息化水平。

六、全面加快改革步伐，提高开放合作实效

突出经济体制改革。以经济体制改革为重点，深化投融资体制改革，研究出台扶持中小微企业发展的投融资政策，积极稳妥推广政府和社会资本合作模式。启动利州、昭化、朝天和青川四县区农村信用社改制组建农村商业银行工作。深化国有企业改革，完成市本级3户企业股权合作，推出5个项目合资合作，加快推进市公交公司等市属国有企业公司化改造。深化农村改革，加快建立三级农村产权交易平台和交易流转体系，加快推进“七权同确”，抓好苍溪县、利州区增加农民财产性收入省级综合试点、昭化区农村小型公共基础设施村民自建省级试点工作。改革财政专项扶贫资金使用管理办法，实施“六大扶贫攻坚行动”，推进扶贫工作项目化、工程化。健全新型城镇化推进机制，完善户籍制度改革实施办法，理顺城市规划管理体制，完善加快推进城镇化进程的政策措施。

突出行政管理体制改革。以行政审批制度改革为突破口，把简政放权、放管结合的改革推向深入。大力简化审批程序，规范行政审批服务，研究制定取消收费许可证后收费管理具体办法。推进财税体制改革，完善县乡财政基本财力保障机制，推进“营改增”以及消费税、资源税改革，推进政府向社会购买服务。整合职能，建立统一的不动产登记机构。按要求稳妥有序推进公务用车改革。

突出文化和社会事业体制改革。深入推进文化体制改革，完善市属国有文艺院团和经营性文化企业法人治理结构，统筹协调新闻媒体对内对外宣传和网络宣传的工作机制，加大传统媒体与新兴媒体融合发展力度。深化教育体制综合改革，做好青川县、利州区义务教育均衡发展省级评估和国家认定工作，落实义务教育免试就近入学政策。深化医药卫生体制改革，全面取消公立医院药品加成，扩大医疗联合体试点，加快推进健康服务业发展。

突出生态文明体制改革。纵深推进低碳城市试点，持续推进低碳载体建设，强力推进省级生态县建设。完善城乡环境建设长效机制，建立市、县区及部门联动机制，建立全域覆盖的城乡保洁机制。

全面扩大开放合作，落实差别化的招商引资政策，充分利用资源优势和产业承接条件，包装推出一批重大项目。积极搭建各类投资促进活动平台，组织参加“中外知名企业四川行”“川欧经济合作洽谈会”等重大投资促进活动，力争新签约项目合同资金500亿元以上，引进到位市外资金400亿元以上。深化港澳台、浙广、九广合作，扩大厅市、市校合作，巩固与对口援建省市的合作，强化与川东北经济区各市、绵阳科技城及其他周边市州的合作，推进嘉陵江流域开发合作。力争开通广元国际口岸机场。加快发展以机械电子、新材料和生物医药等产业为重点的对外加工贸易，努力培育外贸出口骨干企业，壮大外贸主体队伍。

全面促进支持民营经济发展的改革措施落地，加大对大众创业、万众创新的政策支持，不断激发民营经济发展活力。着力推进民营经济转型升级，积极引导个体工商户转型升级为小微企业，大力支持民营企业通过以商招商、扩大生产再投资进入“四上企业”，促进全市民营经济总量扩大、结构改善、贡献提高。

七、更加注重改善民生，推动社会事业全面发展

针对新形势民生领域出现的新情况，更加注重保障基本民生，更加关注低收入群众生活，更加重视基本公共服务均等化，确保一般公共预算民生支出比重稳定在65%以上。继续实施就业促进、扶贫解困、教育助学、社会保障、医疗卫生、百姓安居、民生基础设施、生态环境、文化体育、城乡电网改造“十项民生工程”。在突出办好16件省定民生大事的同时，实施好14件市定民生大事：完成广巴广陕高速公路连接线工程；开工建设广巴广陕高速公路大石互通连接线工程；加快国道108线陵江-宝轮改线工程，达到初通能力；完成北二环四段道路工程；完成陵宝二线工程；完成严家湾桥梁工程，开工建设严家湾隧道工程；开工建设陵江枢纽工程；整治一批背街小巷；建设134座港湾式公交站台；抓好“菜篮子工程”，新建城市调节蔬菜基地3850亩；全面取缔白龙湖网箱养殖，开展白龙湖、亭子湖沿线城乡环境综合治理；开工建设市残疾人康复中心；建立广元农产品网络交易平台；实施好计划生育特困家庭扶助项目。

全面推进社会事业发展。教育事业发展方面，实施好第二期“学前教育三年行动计划”和“特殊教育提升计划”；全面实行“三免一补”，实施好农村义务教育阶段学生营养改善计划；大力实施“名师工程”；加快建设广元天立国际学校和广元万达中学；大力发展现代职业教育，启动四川健康职业技术学院建设，抓好川北幼专高职高专人才培养评估迎检工作，积极支持四川信息职业技术学院创建省级示范高职院校。卫生计生事业发展方面，加强疾病防控，提升卫生应急处置能力；争创全国农村中医药工作先进市；大力实施“名医工程”，加强卫生计生人才队伍建设，提高卫生计生服务管理水平。文化体育事业发展方面，加快建设幸福美丽新村文化院坝，深入推进基层公共文化服务设施建设；推动文艺创作和文化产业繁荣发展；倡导全民阅读，营造书香社会；加强文物考古调查和重点文物保护；抓好档案业务基础建设；加快地面数字电视和应急广播体系建设。促进全民健身、竞技体育和体育产业发展，做好省第十三届运动会、第九届残运会、第四届特奥会筹备工作。大力推进科学技术的普及和应用。认真做好国防动员、民兵预备役、双拥、统计、审计、水文、国家安全、保密、地方志、人防、消防、老龄、妇女儿童、青少年、慈善、工会、残疾人、红十字、外事侨务、港澳台、民族宗教等工作。

健全社会保障体系。完善覆盖城乡居民的社会保障体系，深入开展“全民参保登记计划”，稳步推进机关事业单位养老保险制度改革，继续推进基本医疗保险城乡统筹，完善城乡居民大病保险制度。实施好“十大救助制度”，加强城乡低保规范化、动态化管理。抓好城镇就业困难人员、农村转移劳动力、退役军人就业工作，全年实现城镇新增就业3.2万人。加强养老服务体系建设，提升养老服务水平。完善综合防灾减灾体系，实现省、市、县区、乡镇四级互联互通。

八、加强生态环境保护，提升可持续发展能力

抓好城乡造林绿化和森林资源保护，大力实施天然林保护、新一轮退耕还林等重点工程，新增森林面积10万亩；加强自然保护区、生态功能区、森林公园、湿地公园、国家园林城市建设和管理。深入实施新环境保护法，落实大气污染防治行动计划，提升空气环境质量；实施水污染防治行动计划，综合治理南河、嘉陵江、白龙江等重点流域，加强饮用水源地保护；深入推进城乡环境综合治理，开展农村生活垃圾专项治理行动，完成12个中央农村环境连片整治项目任务，加快城镇污水垃圾处理设施建设。

深入推进低碳发展战略。实施好低碳园区创建、新能源示范市建设、生态人居改善、试点示范引领和基础能力提升“五大工程”。大力开发利用太阳能、水能、风能、生物质能、天然气、沼气等清洁能源，加快“气化广元”进程。引导企业加快低碳技术改造，促进产业结构低碳化。推广普及测土配方施肥等低碳农业技术。开展好世界环境日和国家低碳日活动，倡导低碳消费理念，培育低碳文化。严格落实节能减排目标责任制，抓好重点领域、重点行业、重点企业的节能减排，力争单位生产总值和单位工业增加值能耗分别下降2%、3%，全面完成节能减排任务。积极发展循环经济，大力推进工业废物和生活垃圾资源化利用，力争工业固废综合利用率达96%以上。强化建筑节能监管，大力推广绿色建筑。抓好交通、服务业、机关单位等领域节能降耗，有效推进公共机构节能。开展节能减排装备、技术推广应用示范。

九、深入推进依法治理，维护社会和谐稳定

大力推进依法治市，创新社会治理方式。深入开展“法律七进”活动，建设覆盖城乡的公共法律服务体系。坚持用法治思维和方式化解社会矛盾，着力解决好企业改制、征地拆迁、环境保护工作中引发的矛盾。坚持源头治理，推进城乡社区建设，健全基层综合服务管理平台。认真开展重大决策社会稳定风险评估。不断完善“大调解”工作体系，深入推行“五建五强”群众工作法。继续落实各级领导干部接访、下访、包案化解信访问题机制，加强网上信访工作，依法规范信访秩序。深化社会组织管理改革，加快行业协会学会与行政机关脱钩。支持群团组织依法参与社会治理。

深化“平安广元”建设，深入推进社会治安防控体系建设，切实抓好反恐防暴专项斗争，依法严厉打击各类违法犯罪活动，加强社会治安综合治理。进一步落实“党政同责、一岗双责、齐抓共管”安全生产责任，全方位强化安全生产，重点加强煤矿、危化品、建筑施工等重点行业领域和人员密集场所的安全监管，深化巩固道路交通综合整治工作，坚决遏制重特大安全事故发生。做好地震、气象、测绘、地质灾害防治等工作，提高公共突发事件防范处置和防灾减灾救灾能力。积极创建“无传销城市”，巩固“无毒害市”创建成果。强化重大动物疫病防控，加强农产品和食品药品安全监管，加快建立可追溯制度和质量标识制度，严厉打击不法行为，切实维护人民群众“舌尖上的安全”。推进“质量强市”建设，加强知识产权保护和工商行政管理，营造公平竞争的市场环境。

加强政府自身建设

各位代表，新形势、新任务对政府自身建设提出了新的更高要求。新的一年，我们将全面推进依法治市，加快建设法治政府、创新政府、廉洁政府和服务型政府，不断增强政府执行力和公信力，进一步促进政府依法行政、高效施政、从严治政。 坚持依法行政，推进法治政府建设。坚持法定职责必须为、法无授权不可为，全面履行好法律赋予的职能。健全政府常务会议和部门办公会议学法制度，完善政府法律顾问制度，认真执行重大行政决策法定程序，提高政府依法决策水平。建立健全行政执法责任制，完善行政执法程序，规范行政执法裁量权，深入推进综合执法。加强权力制约监督，完善行政过错责任追究制。坚持以公开为常态、不公开为例外原则，全面深化政务公开。自觉接受人大及其常委会的法律监督、工作监督和政协的民主监督，认真听取人大代表、政协委员，以及各民主党派、工商联、无党派人士和人民团体的意见和建议，主动接受社会监督。

坚持高效施政，切实提高行政效能。经济发展进入新常态，精神面貌要有新状态。严格落实中央、省委和市委关于作风建设的各项规定，巩固拓展党的群众路线教育实践活动成果。建立健全密切联系群众、改进作风常态化制度，大力治理“庸懒散浮拖”等不良现象，坚决纠正不作为、乱作为、慢作为，坚决惩处失职、渎职行为。加强调查研究，改进文风会风，扎实开展“三服务”公开承诺、“百企千村万人”评效能等活动，切实提高机关效能。加强行政决策落实督促检查监督，确保政府重大决策落实到位、执行到位。

坚持从严治政，深入推进廉政建设。认真落实党风廉政建设各项规定，坚持不懈纠治“四风”，联动推进“三早”预警、廉政风险防控、廉勤监督三大机制建设，加强以廉洁机关为重点的廉洁细胞建设。完善债务管控机制，强化“三公”经费管理。加强招投标、政府采购、土地出让、矿产资源开发、专项资金使用等重点领域和关键环节的监管，着力从源头上预防腐败。始终保持惩治腐败的高压态势，严肃查处各类违纪违法行为，坚决查纠损害群众利益的突出问题。建立健全党风廉政教育常态机制，增强干部廉洁自律意识，树立干部清正、政府清廉的良好形象。

各位代表！做好今年的政府工作，任务艰巨、责任重大。让我们更加紧密地团结在以习近平同志为总书记的党中央周围，高举中国特色社会主义伟大旗帜，在省委、省政府和市委的坚强领导下，团结带领全市人民，坚定信心、锐意创新、扎实工作、奋发有为，为实现全面小康和建设“美丽广元、幸福家园”而努力奋斗！
